# The Noncompliant Patient in Psychiatry: The Case For and Against Covert/Surreptitious Medication

**DOI:** 10.4103/0973-1229.58822

**Published:** 2010

**Authors:** K. S. Latha

**Affiliations:** * *Associate Professor, Department of Psychiatry, KMC Hospital, Manipal University, Manipal-576 104, Udupi, India.*

**Keywords:** *Covert medication*, *Surreptitious prescribing*, *Informed Consent*, *Substitute Decision-Maker*, *Best Interests*, *Autonomy*, *Dignity*, *Competency*, *Advance directives*

## Abstract

Nonadherence to treatment continues to be one of psychiatry’s greatest challenges. To improve adherence and thus improve the care of patients, clinicians and patients’ family members sometimes resort to hiding medication in food or drink, a practice referred to as covert/ surreptitious medication. The practice of covert drug administration in food and beverages is well known in the treatment of psychiatrically ill world-wide but no prevalence rates exist. Covert medication may seem like a minor matter, but it touches on legal and ethical issues of a patient’s competence, autonomy, and insight. Medicating patients without their knowledge is not justifiable solely as a shortcut for institutions or families wishing to calm a troublesome patient and thus alleviate some of the burdens of care giving. The paramount principle is ensuring the well-being of a patient who lacks the competence to give informed consent. Ethically, covert/surreptitious administration can be seen as a breach of trust by the doctor or by family members who administer the drugs. Covert medication contravenes contemporary ethical practice. Legally, treatment without consent is permissible only where common law or statute provides such authority. The practice of covert administration of medication is not specifically covered in the mental health legislation in developing countries. Many of the current dilemmas in this area have come to public attention because of two important developments in medical ethics and the law - the increasing importance accorded to respect for autonomy and loss of the parens patriae jurisdiction of the courts [parens patriae means ‘parent of the country’; it permitted a court to consent or refuse treatment on behalf of an ‘incapacity’, or alternatively to appoint a guardian with such powers].

## Introduction: Focus of the Problem & Concerns; Some Usual Scenarios

“You win the battle but lose the war, if the goal is to help the person get better. There are many ways to heal.”

(Ahern & Tosh, 2005)

The practice of nurses hiding drugs in food and drink has been raised publicly by campaigner Hunter Watson, who discovered that staff in a Scottish care home had sedated his mother without consent, and had disguised drugs in her meals. Mr Watson has raised this serious issue with the Scottish Parliament (Scott and William, 1997).

Examine this hypothetical clinical scenario - A young man barges into the Casualty saying he has a bipolar disorder. He admits to having consulted in the same hospital and being prescribed medications but since last two weeks having been off it and complains of homicidal and suicidal ideations. Hospital records show that, on a prior admission, the patients and staff were bashed badly during application of physical restraints. When the patient becomes increasingly agitated, refusing treatment or admission, the Casualty physician offers him a sealed orange juice container into which antipsychotic medications have been injected. Is the physician’s action justified or ethical?

This scenario raises far more questions than it answers. No one is likely to argue that covert medication is superior to treatment with informed consent, but often the so-called consent process is really a show of force followed by physical restraint and medication. The question posed here is controversial because, given this limited information; reasonable people can disagree about the best clinical decision. Not every emergency department is well staffed and equipped to handle psychiatric emergencies, and this action would not be taken unless the physician felt there was no safe and decent alternative.

In response to this case the following focused question was formulated: In patients with mental disorders (schizophrenia, dementia and so on), is use of concealed medications in food or drink, rather than prescribing medications in the usual way or forcibly administering them, ethically justifiable?

There are four parts to this focused question. The first part concerns the patient population, - patients with mental disorders or dementia. The second part concerns the intervention, which is the use of concealed medications in food or drink. The third part is the comparison of this intervention with the alternative of prescribing in the standard fashion, i.e., openly with the patient’s consent, or forcibly provided that a court order can be obtained. Forcible medication involves the physical restraint of the patient and then injection intramuscularly, without the patient’s consent and against the patient’s objections. The fourth part concerns the best interests of the patient, which here concerns whether there is an ethical justification for concealed medication. We will discuss these points as the paper unfolds.

The practice of covertly administering medication is controversial. Although condemned by some as overly paternalistic, others have suggested that it may be acceptable if patients have permanent mental incapacity and refuse needed treatment. ‘Ethical, legal, and clinical considerations become more complex when the mental incapacity is temporary and when the medication actually serves to restore autonomy’ (Wong, Poon and Hui, 2005).

We discuss these issues in the context of a young man with schizophrenia. His mother had been giving him antipsychotic medication covertly in his tea. Should the doctor continue to provide a prescription, thus allowing this to continue? We discuss this case based on the “four principles,” ethical framework, addressing the conflict between autonomy and beneficence/non-maleficence, the role of antipsychotics as an autonomy restoring agent, truth telling, and the balance between individual versus family autonomy. This framework of the “four principles of biomedical ethics” is as proposed by Beauchamp & Childress (1994), (1) the conflict between the principle of autonomy and the principles of beneficence and non-maleficience; (2) the special consideration of the use of antipsychotic medication that restores mental competence and autonomy; (3) maintaining a balance between family autonomy and individual autonomy, and (4) upholding the principle of truth telling, which is based on respect for autonomy.

## Covert Medication and surreptitious prescribing-Concepts, Meanings and Clarifications; Covert Medications in different populations and across different conditions

### Covert Medication and Surreptitious Prescribing- Conceptual clarifications

Non-adherence by psychiatric patients remains a challenge. Estimated rates of non- adherence among all psychiatric patient groups range between 20 and 50%, and rises as high as 70 to 80% among patients with schizophrenia (Breen & Thornhill, 1998). To improve the care of patients with severe mental illness, clinicians and family members sometimes resort to concealing medications in food or drink--a practice referred to as Covert Medication.

Psychiatric practice is more vulnerable to criticism than any other area of medicine (Mason & McCall Smith, 1999). The interventionist philosophy of psychiatry and the legacy of the psychiatric practices of the past century have left a notch on society’s mind -- of coercive treatments against a background of unscientific evidence of benefit, while other disciplines of medical specialties are spared. Public attitudes, including stigma and suspicion, make it more difficult for psychiatry to claim a similar defense, and modern psychiatrists appear to bear the indelible scars of their predecessors’ actions. The practice of illegally administering medication to patients suffering from psychotic disorders or to patients who refuse or resist treatment has had virtually no mention in the development of psychiatry. Covert/concealed medication, sounds like a coercive remnant from a notorious past, a reminder that institutional practices are still alive and well into the 21st century.

### Covert medication

Covert medication is the practice of hiding medication in food or beverages so that it goes undetected by the person receiving the medication. Pills may be crushed or medication in liquid form may be used (Griffith 2003). This practice exclusively applies to individuals who are not capable of consenting to treatment. It is intended to ensure that individuals refusing treatment as a result of their illness will have access to effective medical treatment. Those who are in favor of this approach argue that it is far less intrusive than administering injectable medication by physically restraining a person who does not want to be medicated. Studies have suggested that around 70% of staff working with vulnerable patients have faced the dilemma of whether they should give medication covertly. Almost all felt that the practice was justified “on some occasions”(Valmana & Rutherford, 1997).

A relatively recent survey by Treloar and colleagues (2000) found that the strategy of hiding medication in foodstuffs was used in 71% of 34 inpatient and residential settings that cared for patients with dementia in England; Srinivasan and Thara (2000) report that it is a common practice for schizophrenia patients at their clinic in India. Some suggest that covert medication should be seen as an emergency procedure (Valmana & Rutherford 1997) rather than a routine, and nurses should talk to other members of the team and patient’s relatives and carers before going ahead with their decision. Others think of it as a means of daring to care, while doing no harm (Singh, 2008).

### Surreptitious prescribing

Surreptitious prescribing is the practice of supplying a prescription to a family member or health care professional of a patient and knowing that the medication is likely to be concealed in food or drink and administered to the unknowing patient. This practice exclusively applies to individuals incapable of consenting to treatment. It is intended to ensure that individuals refusing treatment as a result of their illness/psychopathology will have access to effective medical treatment.

The words chosen “surreptitious,” “insidious” and deceitful practice” is fraught with potential for bias that causes “irreversible damage” in the absence of empirical evidence that would support such a judgment (McCullough et al. 2004). The term “covert treatment” is preferable to “surreptitious treatment or prescribing”; they should not be used synonymously, the latter term being reserved for those cases where there is malafide intent (Singh, 2008).

### Covert medications in different populations and across different conditions

Medical treatment is often given without consent in emergency for life-threatening situations (Valmana & Rutherford, 1997). In pediatric circles, for example, there is a precedent for drugs being administered covertly (or surreptitiously), a practice accepted by both clinicians and parents (Griffith & Bell, 1996). Although covert treatment is not well described in the psychiatric literature (Treloar et al. 2000), it is, nevertheless, more common than one might imagine. In a study of 50 elderly patients, 79 % received their medication surreptitiously. For patients with dementia this figure was 94 % (Treloar et al., 2000). In a survey of 21 psychiatrists, 38% admitted to having participated in surreptitious prescribing (Valmana & Rutherford, 1997). This figure is likely to underestimate the true practice, because many respondents felt uncomfortable on direct questioning about admitting to deceiving their patients.

Fear of professional censure results in minimal discussion or recording in patients’ case notes, which serves to compound the atmosphere of secrecy and suspicion (Kellet, 1996; Welsh & Deahl, 2002).

## Covert Medication and Secretive Prescribing

The Case in Favor of and Against Covert Medication & Secretive Prescribing; The Irreversible Damage Caused by Surreptitious Prescribing

### Covert medicine administration and secretive prescribing: ‘Win the battle, but lose the war’

### A case in favor of covert medication & surreptitious prescribing:

- Covert medication [or ‘surreptitious’ prescribing] has a number of potential advantages in treating patients suffering from severe mental illness. Serious clinical risks and substantial costs are associated with delay in treating patients with acute psychiatric illness (Kelly, 2002).
- The harmful effects of untreated psychosis are well documented (Loebel et al. 1992; Norman & Malla, 2001). Delaying psychiatric treatment among such patients is associated with increased morbidity and poorer outcomes in terms of prolonged individual suffering, increased risk of self-destructive behavior, deterioration of the therapeutic alliance, and increased physical assaults by the patient.
- Serious delays in medical care may occur if rational negotiation or a show of force fails to persuade the patient to cooperate. For instance, delay in initiating treatment of patients with acute psychiatric illness can lead to the demoralization of health care professionals and redirection of limited clinical resources to non therapeutic activities. Covert or ‘surreptitious’ prescribing raises the possibility of intervening at an earlier stage before relapse and the need for certification and admission to the hospital.
- If the patient is delirious rather than psychotic, expedited medical evaluation and treatment may be critical. Similarly the agitated psychotic or delirious patient may not be competent to give explicit consent; the refusal of such consent may constitute evidence of incompetence.
- Withholding medication might be considered a deprivation of the patient’s right to prompt medical and psychiatric stabilization.
- Covert administration of drugs and ‘surreptitious’ prescribing can also prevent the need to repeatedly restrain and forcibly administer injections to patients. Family and caregivers often find this form of prescribing more satisfying, because it may also reduce the need for certification and the use of seclusion and restraint.
- In the case of patients with dementia who forget to take medication because of cognitive decline, restraint can be viewed as a cruel substitute for covert or ‘surreptitious’ administration (Treolar et al. 2001).
- A significant evidence base exists for family involvement in the management of psychotic illness (Sellwood et al., 2003; Pilling et al. 2002; Loebel et al. 1992; Norman and Malla, 2001; Singh, 2007b) and covert or ‘surreptitious’ prescribing could be viewed as willingness of the family to be more involved in a patient’s care.
- We must of course do no harm, but we must also dare to care (Singh, 2008).

### Case against covert medication & ‘surreptitious’ prescribing

- Patients with insight comply with treatment, and are adherent. Alternatively, those lacking insight have been reported to be highly associated with non-adherence. Patients who deny being mentally ill had higher rates of medication noncompliance than patients with greater insight into their illness (McEvoy et al. 1989). Use of subterfuge runs the risk of denying the patient the opportunity of gaining insight. In some cases, insight improves only after recurrent relapses with the realization by the patient of the relationship between nonadherence and relapse.
- Covert medication and thus ‘surreptitious’ prescribing may serve to reinforce the patient’s view that illness is not present and that he or she does not require further treatment.
- The practice may discourage patients from availing themselves of psychiatric treatment, because some perceive covert or ‘surreptitious’ prescribing as granting too paternalistic a role to psychiatrists.
- Some people view covert medication and ‘surreptitious’ prescribing as a cheap means of managing inadequate staffing levels and thus encouraging untidy practice (Whitty & Devitt, 2005).
- Covert medication raises a set of clinical issues that go beyond ethical concerns. Without informing their family members, patients may consume other medications or psychoactive substances that interfere with the therapeutic effects of the concealed medication and may become problematic.
- Constitutes grounds for claims of ‘criminal battery’.
- Exposes physician and hospital to liability based on claim of civil trespass, especially if there is an adverse outcome.
- May foster future distrust of family members, physicians, and nursing staff.
- Involves misuse of power, breach of trust and confidentiality (when family members are involved in decision-making process) (Whitty & Devitt, 2005). Patients may become angry and refuse treatment after learning that their trust was betrayed. The practice may raise patients’ sense of unreality or paranoia. They may reject further treatment if they feel that the diagnoses were unfounded, or that they have gotten better on their own.
- Cultural norms may be considered in deciding for or against covert administration (Wong, Poon & Hui, 2005).
- Covert or ‘surreptitious’ prescribing runs the risk of overlooking research and not improving our understanding of why patients are noncompliant in the first place.
- Patient, doctor, medication, and illness factors are associated with poor compliance, and ultimately our goal should be to better understand the reasons behind noncompliance and address these reasons rather than resorting to covert or ‘surreptitious’ prescribing.

### Irreversible damage caused by covert or ‘surreptitious’ prescribing

- The likelihood of recovery, treatment, is diminished as compliance is doubtful.
- Breach of trust between a physician and patient
- “A potential form of patient abuse” (Treolar *et al*. 2001)

## Ethical Considerations - Capacity and Consent; who takes the decision?

Need For a Substitute Decision-Maker; Competency and the Right to Refuse Treatment; Autonomy, Dignity and Accountability; Doctrine of informed consent.

### Ethical considerations

Is it ethical to hide medication in the food of someone with a dementia such as Alzheimer’s disease, or of an acutely psychotic patient? Should all covert drug administrations be medically sanctioned and recorded and just how common is it for psychiatric hospitals to conceal medication?

The covert administration of drugs in a person’s food or drink has supporters as well as critics. If you talk to professional caregivers they can often give you very good examples of times when concealed medication seemed sensible and logical. At other times giving medication without the approval of the person taking it seems wrong and recording practices tend to back up that assertion. A comprehensive research project of nursing homes in Norway found that the practice of covert drug concealment was common practice, but poorly documented and arbitrary (Kirkevold & Engedal, 2005).

The central ethical issue in this population is whether an adult with the capacity to understand the implications of having a medication prescribed has the right to know what is being given. The finding of incompetence does not preclude an individual’s having the capacity to recognize that he or she is being given a medication that has the potential to cause adverse effects– which creates a dilemma if the patient develops side effects. Does the patient have a right to know about these side effects and the fact that they are caused by a medication? A physician has an ethical obligation to explain side effects to patients, even to patients deemed incompetent. A patient’s discovery of the fact that medication is being given surreptitiously could have devastating effects on the treatment relationship and the patient’s ability to trust the psychiatrist and the treatment team (Ahern & Tosh, 2005).

### Capacity and consent

All treatment requires informed consent. There is a presumption that all patients have capacity unless demonstrated otherwise. A capable individual has the right to decline treatment, even if this decision may negatively affect his or her health or otherwise reduce his or her life span. Patients with capacity must be able to:

- Understand in simple language what treatment is recommended, its nature, purpose and why it is being proposed.
- Understand its principal benefits, risks and alternatives
- Understand in broad terms what will be the consequences of not receiving the proposed treatment.
- Retain the information long enough to make an effective decision.
- Make a free choice i.e. free from pressure (Grisso & Appelbaum, 1998)

Generally a competent adult has the right to refuse treatment, even if that refusal may adversely affect them. An unwise decision must be respected if the patient has capacity. No one else can give consent for an adult, someone over the age of 18 or 16 in some circumstances.

### Who takes the decision? - Need for a substitute decision-maker

Person incapable of consenting to treatment, a substitute decision-maker (SDM) interested in his/her welfare will make a treatment decision on his behalf. In this process one needs to take into account his wishes before he was found incapable. However, if this was not expressed in advance - ‘Advance Directives’ - any wishes regarding treatment decision should be based on best interest of the patient.

### Factors to be considered for an informed decision by SDM-

1. Nature of the treatment: what it is and what it involves
2. Expected benefits of the treatment
3. Potential or likely risks of the treatment
4. Potential or likely side-effects of the treatment
5. Other treatments or therapies that may be available
6. What could happen to the patient if the treatment is not given

There are, however, many instances in which medication is given without consent, covertly or otherwise, in all vulnerable patient populations whose “best” interests are being decided by a variety of surrogates. Examine this “People with dementia are too frequently given powerful sedative and antipsychotic drugs” which make life easier for the caring staff. This may not be in the best interests of the patient. This practice amounts to “a potential form of patient abuse” (Treolar et al. 2001), while it was suggested by the proponents that covert medication should be regulated.

### Competency and the right to refuse treatment

As noted earlier, a competent adult has the right to refuse treatment, even if that refusal may adversely affect them. An unwise decision must be respected if the patient has capacity. No one else can give consent for such an adult, who is 18 years and above.

For patients detained under the Mental Health Act, assessment should be considered appropriate for a person with a mental disorder who requires treatment for that illness but who is refusing that treatment. Lack of capacity may be enduring, temporary or fluctuating. Assessment of capacity should be fully documented in the person’s case-notes and repeated as necessary.

If someone is found lacking capacity, any decision or action taken must demonstrate that the actions or decisions taken have been in the patient’s best interests and treatment administered in the least restrictive fashion. However, there are times when very severely incapacitated patients can neither consent nor refuse treatment. In exceptional circumstances, this may require the administration of medicines within foodstuffs, when the patient is not aware that it is being done.

Consent to medical treatment is probably the most significant principle underlying the law relating to treatment of psychiatric patients. To force medical treatment upon a patient is likely to contravene the prohibition against inhuman and degrading treatment under the Protection of Human Rights Act 1993, which was enacted in India on 8 January 1994, to provide for the constitution of a National Human Rights Commission (NHRC). Sometimes there is question about their right to autonomy or self determination e.g. in cases where they are not informed or have not consented for treatment of, say ECT, and any side effects of any drugs.

When a patient lacks mental capacity and is thus unable to refuse or consent to treatment, covert administration of medication may be lawful, provided-

1. It would be in the view of a reasonable body of medical opinion necessary to use this means to save the patient’s life or prevent deterioration in his health; and
2. Accords with the best interests of the patient. If there is any doubt about the patient’s capacity then a second opinion should be sought, in the usual way (Whitty & Devitt, 2005).

### Autonomy, dignity and accountability

The key importance of respecting the autonomy of individuals who refuse treatment should be recognized. However, this cannot be followed in patients who are disturbed and refuse medicines and, therefore, there is a tendency to resort to covert administration. As a result, professionals are concerned as they are nonadherent to a code of practice, often being disturbed by thoughts such as ‘Are we on the right track?’ They may search their professional ethics, or the wider field of moral philosophy, for clear answers to the ethical problems that arise in clinical practice. [Or, are we simply wrong in believing that there always are clear answers?]

Can the practice of disguising a person’s medication such that he or she is unaware of its administration ever be justifiable by appeal to principles of beneficence and non-maleficience in incapacitated patients, or to concepts of least restriction to person’s freedom and action? What benefits versus harm could result from the ‘tablet in tea’ scenario?

The key ethical principles relating to the use of concealed medication are autonomy, justice, beneficence, and respect for persons. Autonomous patients are presumed to be able to make decisions. Although autonomy is a fundamental principle underlying health care, it must be balanced by the need for public safety and ideals of beneficence and duty to provide care.

In my assertion, these ethical issues concern mainly whether concealed medication violates patient autonomy and undermines trust in the physician-patient relationship. Patients with progressive dementia or inadequately treated major mental illnesses often lack the capacity to make decisions. They may not be able to pay attention, to absorb, retain, and recall information, to reason from present events to future possible circumstances, to appreciate relevant clinical information, and to assess that information in terms of its value and belief, although they may usually be able to say “no” or to physically resist medication. In such cases, to assert that respect for the patient’s autonomy creates an inevitable constraint on what otherwise would be behavior that is deceitful misunderstands the implications of this ethical principle. This has implications for the trust-argument, because patients with significantly impaired decisional autonomy lack the cognitive ability to appreciate a trusting relationship in the first place.

Singh (2008) calls such patients ‘insight-unconscious’. In other words, an autonomy-based objection to concealed medication, which was the most frequent objection but not an argument made, does not succeed. Moreover, the great reverence afforded to individual autonomy and independence in developed societies may not generalize to cultures in developing countries where familial interdependence is stronger and collective goals of the family are dominant.

The issue of validity must also be considered in the concealed medicine debate because persons with mental disorders are not treated justly if they are deprived of fairness, including due process. Beneficence in the pursuit of an individual’s “best medical interests” is the likely rationale for using concealed medicines for a person who does not take them voluntarily. Respect for persons, however, requires that the dignity of a person be respected even when his or her autonomy is subordinated to other interests.

### Doctrine of informed consent

In developed countries these ethical principles find their way into the treatment setting through the doctrine of informed consent. The principles of informed consent for medical treatment, well established in the West, are not as clearly established in India (Jacob and Rajan, 1991). The basic requirements for informed consent are that adequate information is provided for the individual to make an informed decision, that the person is competent to make the decision, and that the decision is made voluntarily. Competence requires the ability to understand the relevant information, to appreciate the nature of the situation and its consequences, to rationally manipulate the available information in order to make a decision, and to express a choice (Berg et al., 2001).

Clearly, people given concealed medicines have not been engaged in informed consent. Among the few exceptions to informed consent are true emergencies - therapeutic privilege wherein the physician determines that full disclosure would be harmful to the patient, waiver by the patient, and lack of competence (Berg et al. 2001). The moot point is whether covert medication can be resorted to in such an emergency. Individuals may also appoint others as decision makers when they are unable to make decisions.

The ethical principle of truth telling (veracity) would be violated if the professionals deceive the patient. If the patient does lack decisional capacity professionals must obtain consent from a substitute decision maker. If prior wishes are not known, or are unclear, the professional decisions must be made based on what the person would have wanted and must be made in the best interest of the person in consultation with the family and other healthcare providers.

Troelar et al. (2001) appreciate this logic of respect for autonomy and thus rightly emphasize that the main consideration is a duty to care, which they state mainly in legal terms, but is, more importantly, an appeal to the well-known ethical principle of beneficence. This ethical principle requires clinicians to provide interventions that involve the greater balance of clinical good over harm for patients. The alternatives to concealed medication are non-treatment and forcible treatment. Non-treatment violates the principle of beneficence and therefore professional integrity, and is therefore ruled out. Forcible treatment risks physician and psychological injury that could be serious, long-lasting, and irreversible on a magnitude perhaps greater than these sequelae in the case of concealed medication. Forcible medication involves biopsychosocial harm, where concealed medications involve mainly psychosocial harm, provided that dosing and efficacy are well established. That is, there is a beneficence-based case to be made for concealed medication. The conclusions expressed by Singh (2008) may be worth recall here:

…covert treatment, i.e. temporary treatment without knowledge and consent, is seldom needed or justified. But, where needed, it remains an essential weapon in the psychiatrist’s armamentarium: to be used cautiously but without guilt or fear of censure. However, the psychiatrist must use it very judiciously, in the rarest of rare cases, provided: i) he is firmly convinced that it is needed for the welfare of the patient; ii) it is the only option available to tide over a crisis; iii) continuing efforts are made to try and get the patient into regular psychiatric care; iv) the psychiatrist makes it clear that its use is only as a stop-gap; v) he is always alert to the chances of malevolence inherent in such a process and keeps away from conniving or associating with anything even remotely suspicious; and vi) he takes due precautions to ensure that he does not land into legal tangles later (Singh, 2008).

Preventing abuse through a system of organizational accountability becomes very pertinent. A thorough going beneficence-based case for concealed medication must take the potential psychosocial harm of a practice of concealed medication seriously and seek to prevent it (McCullough et al. 2007). A system of prior review and justification, accompanied by rigorous quality enhancement, may well achieve this important beneficence-based goal.

One way of approaching the problem is through a casuistic (‘case history approach’) perspective. Consider an adult with a delusional disorder who consistently rejects all oral medication. Nursing staff, in cognizance of the prohibition of oral/injectable covert medication, administer such essential medication by suppository on a daily basis. Consider another elderly patient with cognitive impairment who is acutely disturbed and represents a significant risk of harm to him-/herself or to others. Could benefit outweigh harm if the practice of covertly administering antipsychotics and sedative drugs, respectively, were in fact judged to be the least restrictive measure to maximize such a patient’s liberty and dignity (i.e. less than that accorded by suppository, or restraint followed by intramuscular injection)? Which is more, or less, acceptable in a young healthy adult with incapacity versus a frail elderly person? Should that matter, and is the question ethically specific to individual circumstance or subject to generalization?

A few would dispute the moral duty to administer essential cardio respiratory treatments or even insulin to patients of dementia. What about antipsychotics? Should they not come in the same category? As care-givers of those with dementia did not differentiate moral differences between medication for psychiatric disorder and that for physical disorder (Treolar et al. 2000), nor did professionals (Treloar et al. 2001)?

Also, should the behavioral management of the consequences of dementia and delusional disorder be included in the same category of ‘necessity’?

Is there, nevertheless, a case for a continuum of acceptability of mode of administration in all treatment proposals, just as there is a continuum of levels of capacity required for particular treatment measures (‘a capacity… commensurate with the gravity of the situation’)?

What about forcible treatment?

‘Reasonable force can be used to ensure that the patient accepts treatment’(Case law Norfolk and Norwich Healthcare NHS Trust v W, 1996). In a case in the West (case law Re MB, 1997, cited by Welsh & Deahl, 2002) it was decided that ‘the extent of force or compulsion that might be necessary can only be judged in each individual case and by the health professionals. It may become for them a balance between continuing treatments which is forcibly opposed to deciding not to continue with it’. Case law has served to define the breadth of the duty owed by, and power accorded to, professionals who treat patients who lack capacity.

Instead of a complex debate, can we forget the above and seek refuge in deontological principles which focus on the rightness or wrongness of acts by deciding that if treatment is right, does it truly matter how it is given, in coffee or tea or jam or undisguised on a spoon? Or is all covert medication deontologically wrong? Are we taking refuge under the utilitarian perspective of ends justifying means, of maximization of happiness, of greatest good for the greatest numbers, or is that only a comforting shield?

Finally, if covert medication contravenes contemporary ethical practice, can it ever be made ethical by the inclusion of additional safeguards? As with many complex ethical issues in law and medicine, there are no absolutes and no comfortable reductionist principle that will suit every situation.

## Covert Medication Practice: Mechanism of last resort & Need for Regulatory Mechanisms

The practice of covert administration of drugs seems to be regarded as acceptable for both physical and psychiatric disorders as a last resort (Mason & McCall Smith, 1999) based on what the patient would have wanted and his ‘best interests’. However, what we need to be concerned about is the secrecy surrounding the practice and the lack of regulatory mechanisms, and also the absence of discussion within the treating team including the pharmacists regarding adverse side effects. Moreover, covert medication is often based on the judgment of a single professional, either the psychiatrist or the concerned nurse, and relatives may well be kept in ignorance. This disturbing picture is usually attributed to a culture of fear surrounding the practice in which written guidelines are lacking and concern about getting into wrangles with law drives the practice underground.

From an ethical point of view, covert medication/surreptitious prescribing could be viewed as a form of misuse of power and a breach of trust in the doctor-patient relationship from the patient’s perspective, as the patient is unaware of treatment received. The involvement of relatives and caregivers in the process also raises the issue of breach in confidentiality. These factors may result in irreversible damage to the therapeutic relationship in some cases. Although some may view covert medication/surreptitious prescribing as a deprivation of the rights of the patient, it is also worth remembering that, paradoxically, withholding medication necessary to effectively treat mental illnesses could also be viewed as a deprivation of the patient’s rights.

The ethical principles relevant here are those of autonomy and the duty of care. On face value, the principle of autonomy implies that all deception is wrong, even if serious harm might arise from a refusal of care. However, to seek consent from the incapacitated is futile. If we insisted on consent patients might suffer from being denied care they could not validly reject.

As with many uncertainties in medicine, we often look to the law to guide us through such conflicting moral imperatives, and case law can, in certain circumstances, serve to highlight and clarify the legal position in similar issues presented to the judiciary.

## Legal Considerations

Legally, treatment without consent is permissible only where common law or statute provides such authority. The Indian Mental Health Act 1987, for example, states that being a voluntary patient, who has given his consent in writing or where such person (whether or not a voluntary patient) is incompetent by reason of minority or otherwise, to give valid consent, the guardian or other person competent to give consent on his behalf, has to give his consent in writing for treatment/ research. Akhtar et al. (1998) as well as Nambi (1996) have raised questions on issues related to not having any mention of ‘competence’, ‘consent’ ‘presumption of global incompetence’ and so on in the Act. These terms they believe are rightly to be dealt with in general laws and it is not necessary for us to dissect out all these issues for the day-to-day application of the Indian Mental Health Act. At this juncture it may be enough to revert to definitions, wherein a mentally ill person means a person who is in need of treatment; and when such a person is admitted as per Section 19, it is the psychiatrist’s duty to administer him treatment. The bonafides of treatment that are given could be made transparent by recording in the case files the reasons leading to a particular decision and also getting other colleagues involved in certain complex and important decisions. Even with Special Section 92 in the Act, to protect psychiatrists for actions taken in good faith, it is quite unwarranted for professionals to be unduly concerned about the risk of punishment as apprehended by Akhtar (1990).

The Indian Mental Health Act 1987 Chapter 8 deals with the Protection of Human Rights of Mentally ill Persons. Section 81 (1) states that ‘No mentally ill person shall be subjected during treatment to any indignity (whether physical or mental) or cruelty’. Patients being placed totally under the care of treating doctors are something unique to psychiatry. And neither these patients (because of their illness) nor their relatives who are not allowed to be present are in a position to protect their basic rights. To say that there is no need to safeguard the patient’s interest by suitable statutory measures even in this situation of extreme vulnerability would appear less than fair.

The stance on the legal front is that ‘in the interests of the health and personal safety of that person (mentally ill) or for the protection of others it is necessary to detain him in a psychiatric hospital or psychiatric nursing home for treatment’ (Indian Mental Health Act, 1987) but no mention of covert medication might be implied from this.

## Limitations of Covert or ‘Surreptitious’ Prescribing

Many of them are related to legal issues. The major risk for clinicians who prescribe medication covertly or ‘surreptitiously’ is that they are in effect taking the law into their own hands. One must question whether this form of prescribing in psychiatric care is necessary or legally defensible given the legal methods for involuntary committing and treating patients--involuntary hospital admission, outpatient commitment, and appointment of a guardian--that are outlined in mental health legislations.

Further, antipsychotic medications are associated with well-documented side-effects, including extra pyramidal movements and sudden death in some circumstances.

Malpractice suits against doctors and health care facilities and product liability suits against manufacturers of antipsychotic drugs have taken place in the West among patients who developed tardive dyskinesia as a result of taking antipsychotic drugs. Certain jurisdictions believe that a doctor who proceeds without consent will be liable for trespass, assault, or battery, regardless of whether the doctor believed that what he or she did was good for the patient. In such cases the doctor could be prosecuted as an accomplice to battery.

Covert or ‘surreptitious’ prescribing has the legal implication of a relative acting as the proxy decision maker for a patient without mental competence. Its legality varies across countries. For example, in the United Kingdom relatives do not have such powers, except in Scotland.

## Can Psychiatric Documents Aid Mentally Ill in Crises?

Psychiatric advance directives: reconciling autonomy and non-consensual treatment; Psychiatric Advance Directives: ***Ulysses contract” or “self binding contract”;*** Advance directives: ***Utilities;*** Will psychiatric advance directive be legally binding? ; Have any courts upheld the validity of psychiatric advance directives? ; Case of ***Hargrave v. Vermont;*** do psychiatric advance directives have moral authority? Is there need to have to appoint an agent? Where is this leading us? How to prevent misuse and abuse?

### Psychiatric advance directives: reconciling autonomy and non-consensual treatment

Advance directives have been one of the more promising innovations in recent years to give patients a greater voice in their psychiatric treatment (Appelbaum, 1991).

Advance planning of treatment for mental illness by way of written advance directives is an issue of debate in contemporary mental health care globally (Brock 1993; Srebnik & Fond 1999). Advance Directives, also known as a ‘living will’, enable a competent person to make decisions about future treatment, anticipating a time when they may become incompetent to make such decisions.

### Psychiatric advance directives: “ulysses contract” or “self binding contract”.

The general model of a psychiatric advance directive is the so-called “Ulysses contract” or “self binding contract”. Different authors have proposed such contracts as instruments of “consent-in-advance” or “advance treatment authorization” (Brock 1993; Lavin 1986). The name “Ulysses contract” refers to Homer’s example of Ulysses instructing his crew to bind him to the mast of his ship before they sailed past the irresistible sirens, and to ignore his requests for release. Thus he was able to enjoy the beautiful singing of the sirens without suffering the disastrous results that would normally have followed. Singh (2008), for example, has no hesitation in making such an advance directive permitting covert treatment being administered to him if the need arises.

Advance directives for psychiatric care are the subject of debate in a number of Western societies. In English law, if “clearly established” and “applicable to the circumstances”, an advance directive assumes the same status as contemporaneous decisions made by a competent adult. A psychiatric advance directive, anticipating relapse of a psychosis, develops the concept of the living will. It can be argued that it could reconcile two apparently contradictory themes in the current practice of psychiatry – on the one hand, the call to provide for non-consensual treatment outside hospital, and on the other, the promotion of patient autonomy (Halpern & Szmukler, 1997).

One of the earliest proponents of advance directives, Thomas Szasz–a fierce critic of psychiatric diagnosis and treatment suggested that people with mental disorders use advance directives to preclude future treatment, especially treatment with medications. This is diametrically opposite to the stand an advance directive can mean for Singh (2008). As Szasz (1982) saw it, if advance directives represented the unalterable choices of competent patients, there would be no way to override the preferences embodied in the directives. Singh (2008), on the other hand, sees it as a means by which a person, when competent, decides what is to be done for him when he becomes incompetent.

### Advance directives: Utilities

- Allow patients to appoint proxy decision makers
- To make choices about particular treatments
- To take effect should patients become incompetent to make decisions for themselves
- Encourage patients and clinicians to discuss future contingencies
- To negotiate mutually acceptable approaches to care

### Will psychiatric advance directive be legally binding?

While advance directives for healthcare have been around a long time, their use for psychiatric care is a very new area of law. We do not yet know how courts will deal with them, especially when safety issues arise.

All states in the West have statutes that govern the use of advance directives, which can be applied to general medical and psychiatric care, and many states now have special provisions for advance directives for psychiatric care per se.

### Have any courts upheld the validity of psychiatric advance directives?

Permitting people who are not mentally ill to engage in advance planning through advance directive instruments on a wider basis than people with mental illnesses raises significant issues. To date one federal court in the US has addressed such an issue. A Vermont law allows doctors to go to court to nullify mental health provisions in a durable power of attorney/advance directive if the treatment choices made by the agent do not result in improvement of the declarant’s condition.

### Case of Hargrave vs. Vermont

The case, Hargrave vs. Vermont, grew out of a complaint filed in 1999 on behalf of Nancy Hargrave, a woman with a history of paranoid schizophrenia and multiple admissions to the Vermont State Hospital (Hargrave vs. Vermont, 2003). Hargrave had completed an advance directive–known in Vermont as a “durable power of attorney for health care,” or DPOA–in which she designated a substitute decision maker in case she lost competence and in which she refused “any and all anti-psychotic, neuroleptic, psychotropic, or psychoactive medications.” The major national law firm that represented Hargrave immediately filed suit to block the State of Vermont from overriding her advance directive should she ever again be involuntarily committed and obtained certification to represent the entire class of patients in similar situations. Hargrave’s target was Act 114, a 1998 Vermont statute that attempted to address the dilemma inherent in psychiatric advance directives. Although advance directives were intended to facilitate patients’ participation in treatment decisions, they have, as noted, the potential to prevent all treatment, even of patients who are ill enough to qualify for civil commitment under the prevailing dangerousness standards. To mitigate this prospect, the Vermont legislature allowed hospital (or prison) staff to petition a court for permission to treat an incompetent involuntarily committed patient, notwithstanding an advance directive to the contrary. Before the court could authorize nonconsensual administration of medication, it had to allow the terms of the patient’s advance directive to be implemented for 45 days. So a patient like Hargrave, who had declined all medications, would be permitted to go unmedicated for a 45-day period, after which the court could supercede the patient’s refusal of treatment.

The core of Hargrave’s challenge to the statute was based on Title II of the Americans With Disabilities Act (ADA), which requires that “no qualified individual with a disability shall, by reason of such disability, be excluded from participation in or be denied the benefits of the services, programs, or activities of a public entity, or be subjected to discrimination by any such entity”

In response, the State of Vermont offered three arguments.

- Because Hargrave had been involuntarily committed, Vermont claimed that she qualified under exclusion to the ADA for persons who pose a “direct threat.”
- The State contended that the plaintiff was not being discriminated against on the basis of disability, because anyone who completed an advance directive was susceptible to having his or her choices superceded.
- In any event, it was the status of being civilly committed, not being mentally ill that was the point of distinction here.
- Vermont looked to a federal regulatory provision that allowed a public entity to continue existing practices, despite an ADA challenge.

### Do psychiatric advance directives have moral authority?

The moral authority of advance directives can be based upon the principle of respect for patient autonomy. It is just not adherence to patient’s wishes but the values that the patient endorses.

### Is there a need to appoint an agent?

That depends on the law in the state. Some states may set up an advance directive without appointing a person to act for the patient.

### Where is this leading us to?

The ultimate scope and impact of Hargrave may not be known until a decade from now; it is worthwhile to consider the possible effect of the decision on the use of advance directives for psychiatric treatment. Current research suggests that most patients who complete advance directives do not use these directives to decline all treatment with medication, but rather to indicate preferences among alternative treatments, or to inform future treating doctors of particular concerns–for example, the care of their pets or children while they are hospitalized. Although Hargrave may fortify some enthusiasm for advance directives among patients who are opposed to receiving any medication, it remains to be seen how common the phenomenon will become.

Studies are now under way that will tell us more about the utility of advance directives in psychiatry–for example, whether, given the current state of the mental health system, advance directives actually have an impact on subsequent care. At a minimum, however, it seems likely that Hargrave, as it becomes more widely known, will chill enthusiasm for psychiatric advance directives among many clinicians. Examples of case law and statutes explicitly support the involuntary administration of medications contrary to an individual’s express desire to refuse treatment.

### How to prevent misuse and abuse?

Advance directives may become instruments of power and control in the hands of mental health professionals.

Some solutions would be:

1. Permitted only when the individual’s illness was recurrent, interspersed with periods during which behavior was relatively symptom-free.
2. Required that the person involved had experienced a specified number of psychotic episodes in the past, and contracts would be permitted only when the individual’s disorder was responsive to treatment
3. Contracts would be drawn at the initiative of the patient to avoid the exercise of coercive influence by psychiatrists
4. The patient’s disorder be in remission at the time the contract is made
5. The individual’s legal competence at the time of the contract formation would have to be established
6. Cautious about third party involvement (with malicious intent) to ensure that the patient’s best interests are served and preserved
7. Contract be valid for a limited time
8. Patients could renegotiate or revoke the agreement at any time other than during a relapse as defined in the contract
9. Contract-sanctioned commitment and/or treatment would be allowed to continue for only a short time
10. Court involvement to remain a right of the patient

The major practical problem about directives concerns the relationship between advance authorization and access to psychiatric treatment and care. Can the wished treatment be provided when the patient is in crisis? Is there any guarantee that a specific care facility has the resources to provide care? Will patients indeed perceive Ulysses contracts as instruments that promote shared power between themselves and their doctors? Empirical research must show whether psychiatric advance directives in the form of Ulysses contracts have any practical relevance and what their practical shortcomings are.

## Some Guidelines

Stroup, Swartz, and Appelbaum (2002) take a broad approach, considering the roles of the patient, the patient’s family, psychiatrists, and the service system in providing care for patients with schizophrenia. They conclude that psychiatrists should not routinely direct family members to conceal medications. Instead, advance directives and other approaches, including psycho-education, should be considered.

The decision whether or not to administer medication covertly should be considered by the multi-disciplinary team, and it is good practice to consult the family of the patient with regard to such decisions. The clinician responsible for the patient’s care should consider the following points, all of which should be recorded in the notes of the patient:

- Whether the patient is competent to consent to or refuse treatment?
- Why is it proposed to administer medication covertly?
- If a patient is incompetent, whether it is necessary to save a patient’s life/prevent deterioration in his health, and it accords with his best interests.
- Whether, in the case of an incompetent patient, the patient is likely to recover so as to be capable of making his own treatment decisions in the near future.

On the contrary, the position is more difficult if a patient has capacity. A person who has been detained under the MHA is not necessarily incapable of giving or refusing consent. The Act does, however, make provision for patients to be treated for their mental disorder without consent in certain circumstances and the use of covert medication may be justified.

## What Next?

Sensible and compelling arguments exist for and against the use of covert medications in many settings (Welsh &, Deahl 2002; Treloar et al. 2001; Ahern & Tosh 2005; Radcliff 2005; Singh, 2008).

**Figure 1:**
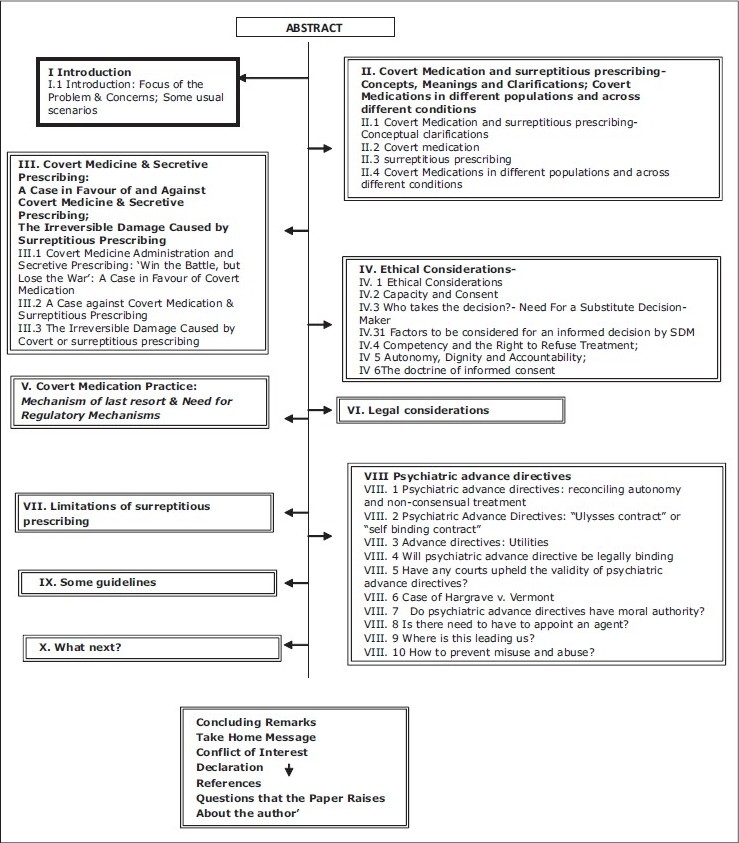
Flowchart of the paper

In summary, it can be concluded that the stance to be taken in our decision on covert medication must be based on clinical evidence. Before considering covert administration of medications, reasonable measures of persuasion should be attempted and only after this has been exhausted should the staff be comfortable with the proposed strategy. Any benefit of covert medication needs to be balanced with the risk of giving medication covertly.

A documented history of relapses, previous injury, or other adverse outcomes related to previous physical restraint may guide in decision-making.

Whenever family members are available, they should be kept informed of the Physician’s intentions, and, if possible, approval from them or consent from a health care proxy should be obtained. Communication lines between the treating team, patients and relatives as far as possible should be kept open, avoiding secrecy (transparency to be safeguarded) in the administration of medicines, with constant feedback. If covert administration of medications does occur, it may be appropriate to inform the patient of the circumstances once he or she is stabilized.

Advanced statements and the person’s past and present wishes should be taken into account. Stable patients with insight should be encouraged to prepare an “advance directive” specifically addressing such situations.

It is important for the nursing and psychiatry staff to clearly understand each other’s priorities and discuss each of the practice methods of patient management without being judgmental. The decision whether or not to administer medication covertly should be considered by the multi-disciplinary team and it is good practice to consult the family of the patient with regard to such decisions. Documentation of covert medication is another issue that has to be looked into. Covert or concealed medication sometimes becomes inevitable, but care should be taken to preserve the respect, dignity and rights of the patient by careful handling.

## Concluding Remarks

It should be clear from the discussion above that the use of covert administration will depend upon a number of variables. Consideration should be given to whether the patient is competent, detained under the MHA or informal; and the basis upon which the use of covert administration is proposed. Providers of healthcare should therefore seriously consider introducing a policy relating to medication administered in this way if one is not already in place. Staff should be given guidance as to the criteria that should be considered when reaching a decision on whether covert medication could be justified; a policy and a set of guidelines can assist in directing the staff through their decisions, and avoid overuse and abuse related to its practice.

### Take home message

The practice of covertly administering medication is universal and controversial but seldom documented as it is carried out in an atmosphere of secrecy. Covert medication and ‘surreptitious’ prescribing has a number of potential advantages as well as disadvantages in treating patients suffering from severe mental illness. Ethical as well as legal issues are inherent in the practice of covert medication with a potential scope for misuse and abuse. Issues related to informed consent and capacity, competency, dignity, autonomy and best interests of the patients need to be looked into and safeguarded. Stringent guidelines need to be drawn with sufficient regulatory controls in implementation.

### Conflict of interest

None declared.

### Declaration

This is an original unpublished work, not submitted for publication elsewhere.

## Questions that the Paper Raises

1. Are radical legislations required to streamline the practice of covert medication?
2. What does one have to say about the consumerism trends and prescription of medicines secretly?
3. With the Human Rights Issues and the RTI (Right to Information) round the corner, can we foresee the redundancy of the practice of Covert or Surreptitious Medication?
4. What are the safeguards/regulatory mechanisms that would make the practice of covert medication more acceptable /useful?

## About the Author


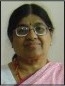
 *K.S. Latha, PhD, is an alumnus from NIMHANS, Bangalore and Roshni Nilaya School of Social Work, Mangalore and National Law School of India, Bangalore. She is an Associate Professor in Psychiatric Social Work at Kasturba Medical College Hospital, Manipal University, Manipal, India. She is also engaged in practice of individual, family and marital therapies, besides being a PhD guide. Her areas of interest in research are psychosocial issues related to suicidal behavior, adolescents in crisis and issues related to women and human rights. She is affiliated to several professional bodies. She has published several articles related to mental health in vernacular in the lay press and has been the recipient of Dr. S.S. Jayram Award instituted by the Indian Psychiatric Society for the same.*
